# Age Estimation Using the Cameriere Methods of Open Apices: A Meta-Analysis

**DOI:** 10.3390/healthcare9020237

**Published:** 2021-02-23

**Authors:** Sorin Hostiuc, Ioana Diaconescu, Mugurel Constantin Rusu, Ionut Negoi

**Affiliations:** 1 Department of Legal Medicine and Bioethics, Faculty of Dental Medicine, Carol Davila University of Medicine and Pharmacy, 042122 Bucharest, Romania; 2 National Institute of Legal Medicine, 042122 Bucharest, Romania; diaconescuioanaa@gmail.com; 3 Department of Anatomy, Faculty of Dental Medicine, Carol Davila University of Medicine and Pharmacy, 020021 Bucharest, Romania; anatomon@gmail.com; 4 Department of Surgery, Faculty of Medicine, Carol Davila University of Medicine and Pharmacy, 020021 Bucharest, Romania; negoiionut@gmail.com

**Keywords:** Cameriere, age estimation, open apices, meta-analysis

## Abstract

Purpose: To evaluate the actual variability of the mean difference between chronological and dental age using the Cameriere method of open apices and to test its accuracy in variable age groups. Method: We selected studies that contained data about the mean, standard deviation, and number of cases for chronological age, dental age and gender. We used a random-effects model. Statistical significance was estimated, at a *p* < 0.05, using prediction intervals. For the analysis of publication bias we used the funnel plot and Egger’s regression test for plot asymmetry. I^2^ was used to test the presence of heterogeneity between studies. The Z test was used to test for statistical differences between subgroups, with *p* < 0.05 being considered statistically significant. We also used 95% for confidence intervals and prediction intervals. Results: In boys, the average difference between chronological and dental age was 0.44 (0.26–0.63) years, while in girls the average difference between chronological and dental age was 0.34 (0.19–0.49) years. In the 6–7 years age group and in the 14–15 years age group, there was a statistically significant difference between dental and chronological age. Our study shows that the Cameriere method is useful for estimating the chronological age, with errors of less than one year. Conclusions: The Cameriere method of evaluating dental age using open apices is sufficiently accurate for forensic practice, at least in the 7–14 age-interval.

## 1. Introduction

Age estimation is one of the most important objectives of forensic anthropology, with wide applicability for cadavers and skeletons, but also for living individuals (to establish age of majority, for adoption, illegal migration, sexual exploitation, or to determine criminal liability) [1,2]. Teeth are especially useful in this regard, mostly in children and adolescents, where the developmental stages are well known and characterized, and they have a decreased variability depending on environmental and genetic factors [3,4,5]. Radiological analysis of dental development is widely used to this intent, being preferred over the morphological analysis of dental eruption, which is more prone to errors (increased variability due to malnutrition, crowding, teeth decay or premature loss [1,3,6]).

In 2006, Cameriere et al. published a method of age estimation based on the measurement of the ratio between the length of the projection of the open apices and the length of the tooth axis major (known as the open apices method). Briefly, the method uses the seven left mandibular teeth. The first step is to identify the ones with closed apices, which are counted, and the sum is abbreviated N_0_. For the rest of the teeth with open apices, the distance between the inner sides of the open apex (for single-root teeth), or the sum of distances between the inner sides of the open apices (for multi-root teeth) is measured. These measurements are abbreviated to A_i_, i = 1–7) and are then divided by the tooth length (L_i_, i = 1–7) in order to obtain the normalized measurements for the seven teeth (x_i_ = A_i_/L_i_). Then, a variable entitled s is computed, which is equal to N0 + sum(x_i_). This value is included in the formula: Age = 8.971 + 0.375g (gender, with 1 for boys and 0 for girls) + 1.631 × 5 + 0.674N_0_ − 1.034s − 0.176sN_0_ [7]. The method was updated in 2007 to Age = 8.387 + 0.282g − 1.692 × 5 + 0.835N_0_ − 0.116s − 0.139sN_0_ [8]. 

Many authors have devised, based on this methodology, methods that have been applied in specific populations (yielding different regression equations [9,10,11,12]), leading to the identification of new areas of interest [13,14,15]. For example, Angelakopoulos et al. developed a new formula—based on the Cameriere approach—using Bayesian methods, in South African children [16]. Additionally, numerous authors have shown this method to be more accurate in estimating age compared to others such as Demirjian, Nolla, Haavikko, or Willems [17,18]. However, the mean difference between chronological and dental age, as estimated by this method, has been shown to be variable in different studies, with some of them having been performed on similar populations [17,18]. Moreover, some studies—which evaluated mean differences in different age intervals—showed variable levels of accuracy depending on the age of the subjects [19,20,21]. However, the statistical significance of these results was not always determined, mainly due to a decreased number of cases per age group. 

The purpose of this study is to evaluate the actual variability of the mean difference between chronological and dental age using the Cameriere method of open apices (both the original and the European formula), and to test the accuracy of the method at variable age groups.

## 2. Materials and Methods

We performed the study according to the PRISMA (Preferred Reporting Items for Systematic Reviews and Meta-Analyses) and MOOSE (Meta-analysis of Observational Studies in Epidemiology) guidelines for reporting systematic reviews and meta-analyses of observational studies in epidemiology [22,23].

### 2.1. Selection Criteria

Inclusion criteria: studies that contained data about the mean, standard deviation, and number of cases for chronological age, dental age, and gender (for the overall values).

We used the following exclusion criteria: (1) the absence of relevant information to obtain the data needed for the analysis; (2) studies with less than 20 subjects; (3) case series/case reports without a specification of the study population from which the cases were drawn upon and without a specific detection algorithm; (4) calculations made using methods other than the original and European formulas developed by Cameriere [7,8]. Two authors independently performed this search. In cases of disagreement between these two authors regarding inclusion of paper in the study and/or the presence of relevant exclusion criteria, a third reviewer was involved and the article was discussed until a consensus was reached.

### 2.2. Search Method

We analyzed the results obtained from Pubmed and Web of Science using the following keyword: “Cameriere”, with a timeframe that ranged from 2005 to 2019. We preferred not to use additional, restrictive criteria (e.g., article type) as other forms of publication (letters, case presentations, reviews) could potentially add relevant data to the meta-analysis (secondary references, discussions, finding other appropriate articles). The reference list of each relevant article was scrutinized for other, potentially relevant studies to be included in the meta-analysis. The references, abstract, and full text were imported in Paperpile.

### 2.3. Data Collection and Analysis

For each study, two reviewers extracted the data separately and included it in Excel Datasheets. We summarized the following information: study, name of the authors, year, total number of cases, country, mean, standard deviation and number of cases per gender/age group, inclusion and exclusion criteria.

### 2.4. Quality Assessment and Risk of Bias

Quality assessment was performed using the checklist from the STROBE (STrengthening the Reporting of OBservational studies in Epidemiology) Statement Checklist for observational studies [24]. This is a 22-items checklist, and the studies were considered to be of low quality if the sum was below 15, average quality if the sum was between 15.01 and 18, and high quality if the sum was between 18.01 and 22. The analysis was performed by two reviewers and the final score was obtained by averaging the results. Risk of bias was included as a point in the above-mentioned checklist and was quantified for each study.

### 2.5. Statistical Analysis

We used Jamovi (1.2, Sydney, Australia) and Microsoft Excel 365 (Microsoft, Redmond, WA, USA) for Mac for statistical analyses. We used a random effects model with the DerSimonian–Laird estimator and raw mean difference for effect size model measures (except for comparing results between groups, where in such cases we used standardized mean differences). Statistical significance was estimated, at a *p* < 0.05 using prediction intervals. For the analysis of publication bias we used the funnel plot and Egger’s regression test for plot asymmetry. I^2^ was used to test the presence heterogeneity between studies, using the following thresholds: 0–35%—most likely not important, 36–55%—moderate heterogeneity, 56–85%—most likely substantial heterogeneity, and 86–100%—significant heterogeneity (average values based on [25]). The Z test was used to test statistical differences between subgroups, with a *p* < 0.05 being considered statistically significant. We used 95% confidence intervals and prediction intervals.

## 3. Results

### 3.1. Search Synthesis

**Figure 1:**
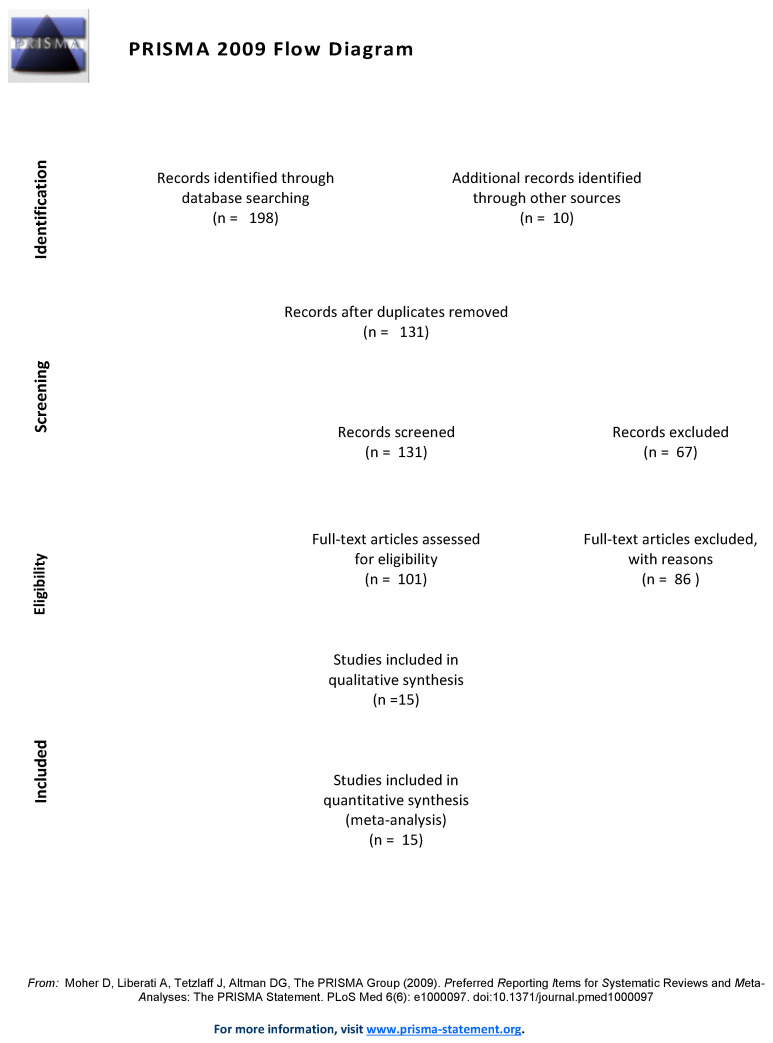
PRISMA flow-chart. Reprinted with permission from ref. [26]. Copyright 2009 the Creative Commons Attribution License. (www.prisma-statement.org (accessed on 17 February 2021)).

During the initial database research, we obtained 116 articles from Pubmed and 82 articles from Web of Science from which, after deleting duplicates and irrelevant studies, we selected 91 to be further scrutinized. By analyzing their references, we found another 10 potentially relevant articles that were also downloaded. From the 101 articles, 15 were included in the final analysis of prevalence. Details about the search synthesis are presented in Figure 1 [26]. The papers contained in the meta-analysis are detailed in Table 1.

### 3.2. Quality Assessment and Risk of Bias

Quality assessment scores were between 13.5 and 20. The score for each study is included in Table 1. No studies showed any significant bias.

### 3.3. Accuracy of Cameriere Formulas Depending on Gender

**Figure 2:**
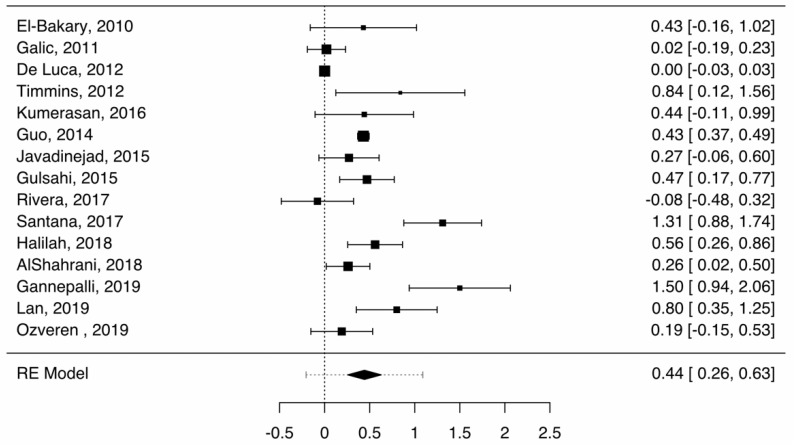
Forest plot—Cameriere, boys, overall (boys).

In boys, the average difference between chronological and dental age was 0.44 years (0.26–0.63), as shown in Figure 2. The heterogeneity was significant (I^2^ = 94.33%). Publication bias was statistically significant (Z = 2.591, *p* = 0.01). In studies using the original formula [11,17,27,29,30,34], the average difference was 0.53 (0.28–0.78). The heterogeneity was most likely significant (I^2^ = 75.02%). Publication bias was not statistically significant (Z = 1.008, *p* = 0.314). In studies using the European formula [19,20,28,31,32,33,35,36,37], the average difference between chronological and dental age was 0.38 (0.13–0.62). The heterogeneity was significant (I^2^ = 87.6%). Publication bias was statistically significant (Z = 2.609, *p* = 0.009). The prediction interval overlapped with the value 0 in all three analyses, rendering the difference between dental and chronological ages not statistically significant. The results obtained using the two formulas were not statistically different (Z = 1.01, *p* = 0.27).

**Figure 3:**
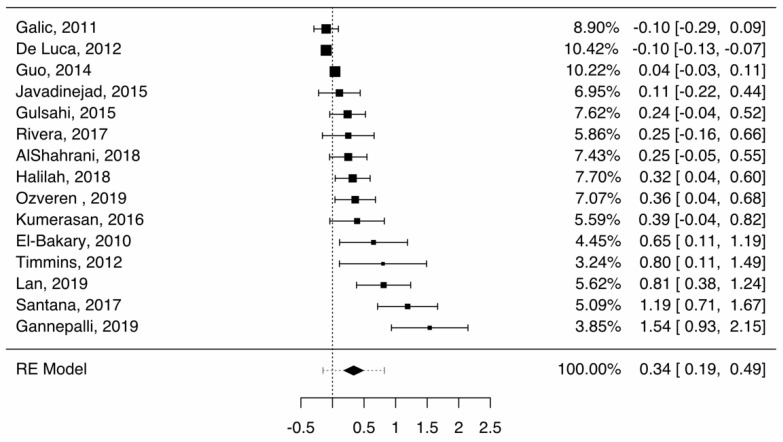
Forest plot—Cameriere, boys, overall (girls).

In girls, the average difference between chronological and dental age was 0.34 (0.19–0.49), as shown in Figure 3. The heterogeneity was significant (I^2^ = 88.83%). Publication bias was statistically significant (Z = 6.464, *p* < 0.001). In studies using the original formula [11,17,27,29,30,34], the average difference was 0.44 (0.11–0.77). The heterogeneity was most likely significant (I^2^ = 84.3%). The publication bias was statistically significant (Z = 2.611, *p* = 0.009). In studies using the European formula [19,20,28,31,32,33,35,36,37], the average difference was 0.34 (0.10–0.58). The heterogeneity was significant (I^2^ = 89.03%). Publication bias was statistically significant (Z = 4.308, *p* < 0.001). The prediction interval overlapped with the value 0 in all three analyses, rendering the difference between dental and chronological ages not statistically significant. The differences between these two methods were not statistically significant (Z = 0.87, *p* = 0.38).

Using the original formula, the difference between boys and girls was not statistically significant (Z = 1.47, *p* = 0.14). Using the European formula, the difference between boys and girls was also not statistically significant (Z = 0.31, *p* = 0.076). As the differences between gender and age formulas were not statistically significant, for the evaluation of the age group accuracy we used combined data.

### 3.4. Accuracy of Cameriere Formulas in Age Groups

**Figure 4:**
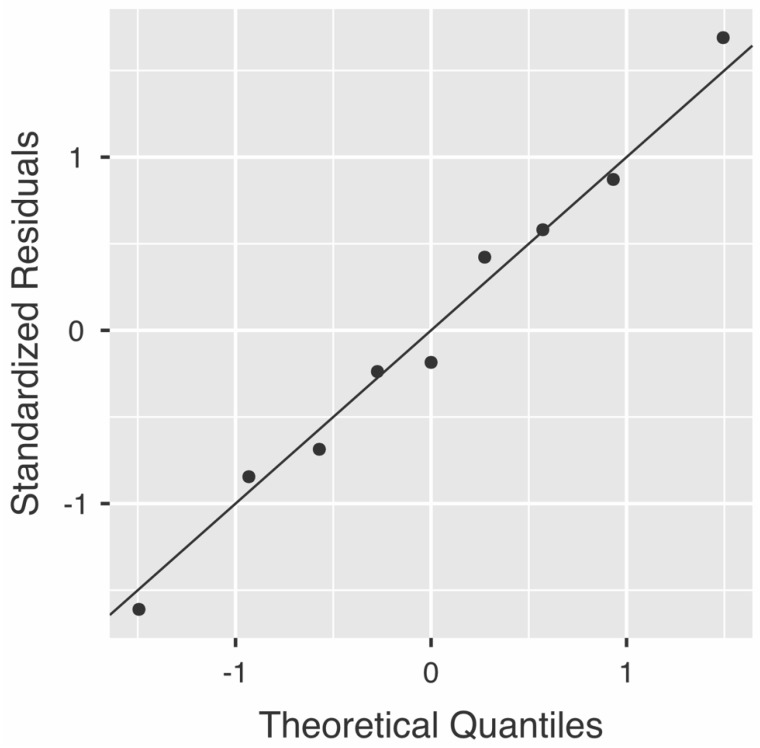
Q-Q Plot. Average differences between age groups.

The results of the analyses performed on age groups are presented in Table 2. In the 6–7 years age group there was a statistically significant difference between dental and chronological age (−0.81, with the prediction interval between −0.05 and −0.71). In the 14–15 years age group there was a statistically significant difference between dental and chronological age (0.87, with the prediction interval between 0.35 and 1.4). The difference between dental and chronological age was not statistically different in the other age groups, but there was an obvious trend, with higher dental ages compared to chronological ages in the younger, and higher chronological ages compared to dental ages in the older subadults (see Figure 4).

## 4. Discussion

Methods based on the analysis of the open apices in teeth have been used extensively in the last fifteen years in order to assess dental age in subadults, but also to evaluate age of majority (based on the analysis of the third molar) [1,2,20] due to their ease of use, high reproducibility and accuracy.

These methods have been evaluated, comparatively, with other dental and skeletal methods of age estimation, and have been shown to have significant advantages. For example, Kumaresan et al. showed that Cameriere’s method was more precise and accurate when estimating dental age in a Malaysian population sample when compared to Demirjian, Nolla, Haavikko and Willems [32]. Javadinejad et al. showed, in an Iranian population aged 3 to 15, that Cameriere’s method was less accurate compared to Smith’s, but more accurate when compared to Willems’ and Demirjian’s [17]. Our study has shown the Cameriere method to be useful for estimating chronological age, with errors of less than a year (and significantly smaller around the 8–11 year range, where the differences between dental and chronological age were minimal). This method has been revised numerous times, usually in order to make it more accurate in specific populations. Rai et al., for example, applied the European formula to an Indian population, showing that the European formula is a significant predictor for age. However, due to geographic differences between different regions from India, they proposed another formula, namely Age = 9.402 − 0.879C (C = 0 for the center and north of India and 1 for the south) + 0.663N_0_ − 0.711s − 0.106sN_0_, which explained 89.7% of the total variance [12]. Cugati et al. developed a specific formula for Malaysian populations, namely, Age = 11.368 − 0.345g + 0.553N_0_ − 1.096s − 0.380sN_0_, which yielded an R^2^ value of 0.871 [18]. Cameriere developed two main formulas to assess dental age based on open apices. The equations are very similar, and they have been used by many authors to evaluate dental age in different populations. Our study has shown that these formulas give comparable results, and this represents the reason why we used studies that were performed using either formula to evaluate the accuracy of age estimation based on the open apices.

In addition to the studies included in our analysis, there have been other studies that used the Cameriere formulas, but the data from such articles was not sufficient to fulfill all the inclusion criteria for this analysis. For example, Cameriere et al., in a study assessing Italian, Spanish and Croatian children aged between 5 and 15, showed the Cameriere method to yield a mean prediction error of 0.407 for girls and a slightly lower value (0.38) for boys [38]. However, the article did not contain sufficient data to estimate the necessary values to warrant inclusion in this meta-analysis.

Wolf et al. compared the usefulness of both the Cameriere and Demirjian methods in a 6–14 year old German population; they gave details about dental age but not chronological age (they only presented the mean difference in various age groups) [39]. The results from this study are in line with ours, showing lower dental compared to chronological ages in the younger age group and a trend reversal after eleven years [39]. Apaydin and Yasar, showed the same trend in a Turkish population, with the Cameriere method overestimating age in younger age-groups (the differences being statistically significant for the age groups 5–6 and 6–7), and underestimating age in older groups (with the differences between dental and chronological ages being statistically significant over eleven years), while the Willems method showed the most precise results. One reason for the age underestimation represents the difficulty in evaluating the small apex opening, which is almost closed/closed in older groups [40]. Timmins et al. showed that the Cameriere method is especially useful up to around 14 years of age (at which point all seven teeth reach maturity), making the Demirjian method more useful in older adolescents (up to sixteen years of age) [29]. Our study showed a similar trend—the only age groups in which the difference between chronological and dental age were statistically significant were the 6–7 and 14–15 age groups. It should be noted that we only evaluated the usefulness of the Cameriere method between 6 and 15 years of age, as the number of studies containing data for younger and older age groups was below four.

The usage of radiological methods for dental assessment in children/adolescents must, however, be used cautiously, as they are considered highly intrusive, due to the risks associated with the procedure, which are not counterbalanced by medical benefits for the patients [41]. Normally, a medical intervention that has more than minimal risks should not be allowed, unless there is a significant medical benefit for the subject and informed consent for the procedure has been obtained. The goal of the procedure is often not for the medical benefit of the patient, but rather for the benefit of a third party, or even maleficent for him/her [42] who can, for example, be deported, or be jailed as a direct consequence of this expertise. Moreover, the issue of consent is highly debatable in many instances—the potential aims of the procedure may be to establish age of majority or criminal competence, as these can be correlated—from a legal point of view—with age. If the subject signs the consent and he/she is not considered legally competent afterwards, to what degree is the consent valid? Similarly, if she/he does not sign the consent, and instead it is signed by a legal guardian, and she/he is considered legally competent, was the procedure not performed without the approval of the person who should accept it [43,44,45]?

Recently, the usefulness of 3D radiological methods for dental age determination have been evaluated, with promising results. Cone-Beam Computed Tomography has been found to be more accurate than orthopantograms for dental evaluation, including dental anatomy and forensics, as shown in a recent systematic review [46]; therefore, its usage might increase the accuracy of the Cameriere method, which could lead to increased usage of the method for dental age assessments.

### Limitations

The first major limitation of this study is the fact that we used two different formulas for estimating the dental age based on open apices. We preferred this approach as the differences between them were minor, and as the initial analysis showed no statistically significant difference between the results of these two studies. However, this approach most likely increased the heterogeneity of the results. Another major limitation is represented by the fact that we were unable to include all studies that were conducted using these two formulas, as the data from some articles were incomplete. The addition of further studies could have yielded more precise results. Additionally, we preferred to use a standardized method for developing the random-effects model (DerSimonian–Laird); this method underestimates the true heterogeneity when the τ^2^ is large and the number of studies is small, but the main advantage of this approach involves the possibility for other meta-analyses—performed with similar purposes—to be compared more easily. The optimal method is that suggested by Hunter and Schmidt (which weights the inverse of the within-study variance), but this is rarely used, and the results would have been harder to compare. Moreover, our initial evaluation (not presented here) showed that the results were highly similar with the published approach. For statistical significance purposes we preferred to use prediction intervals, which are larger than confidence intervals, which are usually used in random-effects models. Our approach decreases the possibility to catch statistically significant differences, however, it is the correct approach to be used from a methodological point of view. Briefly, in a meta-analysis using random-effects models, researchers present the summary effect size and the confidence interval; based on these methods, one can estimate the mean effect size and the precision, but not the distribution of the true effects around the summary estimates [47], which is only correctly depicted by the prediction interval. The age group 3–5 was not included in the analysis due to the very low amount of subject data available. The quality criteria for clinical meta-analyses does not reflect the quality of studies on age estimation. The extent to which the method has been applied correctly or incorrectly in the individual studies was not evaluated. This is particularly important for methods where a large amount of manual measurements are required.

## 5. Conclusions

The Cameriere method of evaluating dental age on open apices is accurate enough for clinical practice, at least in the 7–14 age-interval. It should not be used outside this age range. Its actual usefulness, compared to that of other methods, should be assessed by comparing meta-analyses for each method, using a reproducible methodology. Dental age remains a reliable age estimation with important criminal and civil consequences.

## Figures and Tables

**Table 1:** Summary of studies included in the meta-analysis.

Study	Age Range	Country	No. Subjects	Inclusion Criteria	Exclusion Criteria	Quality Score
El-Bakary, 2010 [27]	5–16	Egypt	286	Age range, good quality radiographs, no agenesis or extractions in the left lower quadrant	Hypodontia, hyperdontia	13.5
Galic, 2011 [28]	6–14	Serbia, Croatia, Bosnia	498	Born after 2000	Systemic disease, premature birth, congenital anomalies, hypodontia of permanent teeth except third molar	17.75
De Luca, 2012 [19]	5–15	Mexico	248	Good quality radiographs, no agenesis or extractions in the left lower quadrant.	Incomplete dental or medical history, evident systemic diseases and congenital anomalies, premature birth, hypodontia of permanent teeth except third molars and hyperdontia.	19.5
Timmins, 2012 [29]	7–17	New Zeeland	200	Healthy children		14
Guo, 2014 [30]	5–15	China	229	Northern Chinese, healthy, no medical pathologies affecting tooth development		18
Javadinejad, 2015 [17]	3–15	Iran	537	Absence of systemic diseases, dental anomalies, nutritional and endocrine problems, premature birth, birth defects, clear birth date and date of radiography		12
Gulsahi, 2015 [31]	8–15	Turkey	573	Good quality radiographs, healthy subjects with known and precise age, no systemic diseases, normal teeth eruption, no pathological conditions associated with the alveolar jaw	Systemic diseases, congenital anomalies, dental anomalies, premature birth, obesity, patients undergoing orthodontic treatments, extraction in the lower left quadrant,	18.25
Kumaresan, 2016 [32]	5–16	Malaysia	426	Malaysian for at least two generations	Radiographs of poor quality, genetic or congenital anomalies, history of orthodontic treatment	18
Rivera, 2017 [20]	6–14	Colombia	457	Patients seeking orthodontic treatment, excellent quality of the ortopanthogram, good general and dental health	Unknown date of birth or date of ortopanthogram, agenesys, hypodontia, missing tooth on the left inside, dental anomalies	16.25
Santana, 2017 [11]	7–17	Mexico	360		Hypodontia, hyperdontia, systemic diseases, congenital abnormalities, evidence of extraction, unclear radiographs	16.5
Halilah,2018 [33]	5–16	Germany	800	Good quality radiographs, children growing up in north Germany, caucasians	Aplasia of at least two corresponding teeth bilaterally in the mandible, extraction in the lower left quadrant, systemic diseases, congenital and genetic anomalies, radiographs with all apices closed	20
AlShahrani, 2018 [34]	6–16	Saudi Arabia	788	Saudi nationality, complete case records	Incomplete medical or dental history, documented tooth extractions or agenesis especially in left lower quadrant, distorted radiographs, radiographic evidence of periapical lesions, fractured teeth and internal tooth resorption, evidence of systemic diseases, congenital anomalies, premature birth, hypodontia of permanent teeth except third molars and hypertonia	14.75
Gannepalli, 2019 [35]	10–15	India	100		inadequate quality for assessment, signs of gross pathology, hypodontia, and previous history of orthodontic treatment were	19
Lan, 2019 [36]	8–16	China	480	Good Rx, no history of drug use or surgery, unaffected teeth, the presence of left mandibular permanent teeth	Maxillofacial malformation, located in Hunan province, no cysts or tumors affecting the development of teeth	18.5
Ozveren, 2019 [37]	6–15	Turkey	636		Systemic diseases, previous restorative, endodontic, orthodontic treatment history, dental trauma history, dental anomalies, missing lower teeth (except the third molar), jaw bone pathologies such as cysts or tumors were	19.25

**Table 2:** Standardized age difference in different age groups.

Age Group	Mean Difference, CI (95%)	I^2^%	Publication Bias Z	Publication Bias *p*	References
6–7	−0.38 (−0.56–−0.21)	67.45	2.599	0.009	[19,20,33,37]
7–8	−0.09 (−0.21–0.04)	43.53	1.969	0.049	[19,20,33,37]
8–9	0.14 (−0.01–0.29)	62.76	−0.908	0.364	[19,20,31,33,36,37]
9–10	−0.03 (−0.26–0.20)	85.74	0.136	0.892	[19,20,31,33,36,37]
10–11	0.16 (0.01–0.32)	81.36	0.850	0.395	[19,20,31,33,36,37]
11–12	0.39 (0.18–0.60)	83.7	0.111	0.912	[19,20,31,33,36,37]
12–13	0.45 (0.08–0.82)	94.91	0.109	0.913	[19,20,31,33,36,37]
13–14	0.56 (0.24–0.89)	94.19	0.276	0.783	[19,20,31,33,36,37]
14–15	0.87 (0.65–1.09)	86.28	−0.826	0.409	[19,20,31,33,36,37]

## Data Availability

None relevant.
